# Treating pediatric OCD with group metacognitive therapy in a naturalistic setting: a preliminary investigation of treatment effects and metacognitive change in light of comorbid disorders

**DOI:** 10.3389/fpsyt.2026.1766674

**Published:** 2026-04-22

**Authors:** Marie Louise Reinholdt-Dunne, Anne Rosenfeldt, Odin Hjemdal, Marie Tolstrup, Henrik Nordahl

**Affiliations:** 1 Private practitioner at Metaminds Psychology Practice, Copenhagen, Denmark; 2 Department of Psychology, Norwegian University of Science and Technology, Trondheim, Norway; 3 Department of Psychology and Behavioral Sciences, Aarhus University, Aarhus, Denmark; 4 Private practitioner at Psykolog Marie Tolstrup, Børne- og unge Psychology Practice, Copenhagen, Denmark

**Keywords:** adolescents, anxiety disorder, children, comorbidity, metacognitive beliefs, metacognitive therapy, neurodevelopmental disorder, obsessive-compulsive disorder

## Abstract

**Introduction:**

Studies suggest that metacognitive therapy (MCT) is a promising treatment for pediatric OCD, but less is known about its mechanisms and effects in light of comorbid disorders. The aim of this study was therefore to explore the magnitude of effects, response and remission rates, and trajectories of change associated with MCT for pediatric OCD in a sample presenting both with and without comorbid neurodevelopmental and anxiety disorders.

**Methods:**

In total, 37 participants aged 9–17 years received eight sessions of group MCT, accompanied by two parental workshops. Participants were diagnostically assessed at pre- and post-treatment, and completed self-report measures before, during, and after treatment.

**Results:**

For the total sample, results showed significant and large reductions in OCD symptoms (Hedges’ *g =* 2.3) and response and remission rates of 81.1% and 51.4%, respectively. Moderate to large improvements were also found in comorbid symptoms, trait worry, and metacognitive beliefs and strategies. When provisionally exploring three types of diagnostic presentations (i.e., OCD only, OCD with comorbid neurodevelopmental disorder[s], and OCD with comorbid anxiety disorder[s]), slightly lower response rates were observed for the two subgroups with comorbid disorders, accompanied by indications of delayed, but steeper trajectories of change in thought-fusion beliefs.

**Discussion:**

Overall, the results indicate that group MCT for pediatric OCD is a feasible treatment in naturalistic settings with high rates of comorbidity, but that the presence of comorbid disorders may be associated with lower response rates and different trajectories of metacognitive change. However, these observations should be considered preliminary and evaluated further in larger samples with more stringent study designs.

## 1 Introduction

Obsessive-compulsive disorder (OCD) is characterized by the presence of intrusive obsessions and compulsions that are distressing, time-consuming, and functionally impairing (1). The estimated prevalence is around 1-3% in both children and adults (2) with more than half of cases having an onset in childhood and adolescence (3). Additionally, 64% of children with OCD also meet criteria for another psychiatric disorder such as neurodevelopmental and anxiety disorders (4). Consequently, there is a need for interventions that effectively alleviate OCD and its underlying mechanisms in children and young people across diagnostic presentations.

Cognitive behavioral therapy (CBT), including exposure and response prevention (ERP), is the recommended first-line of treatment for pediatric OCD (2, 5). Meta-analytic findings indicate a 70% response rate, but only 53% achieve remission following CBT (6), and although the number of comorbid anxiety disorders have been positively associated with outcomes (6), comorbid neurodevelopmental disorders have been associated with poorer outcomes following CBT for pediatric OCD (e.g., 7, 8). Additionally, based on cognitive models of OCD, changes in cognition would be expected to precede changes in OCD symptoms, but while cognitive beliefs have been found to decrease following CBT for pediatric OCD (e.g., 9, 10), changes in them do not appear to mediate treatment effects (10). In fact, changes in OCD severity have been found to predict changes in cognitive beliefs (10), in turn causing doubt around the proposed mechanisms of change (i.e., change in cognitive beliefs).

As an alternative, metacognitive therapy (MCT; 11) proposes a different set of mechanisms by emphasizing the top-down influences on regulation of thinking processes as opposed to the content of thoughts and beliefs. MCT is rooted in the Self-Regulatory Executive Function (S-REF) model (12–14), according to which prolonged psychological distress and disorder are maintained by a perseverative and inflexible style of thinking and behavior (i.e., the cognitive attentional syndrome; CAS) that is activated and regulated by biases in metacognition (e.g., knowledge or beliefs about cognition, internal states, and coping strategies). Grounded in this theoretical framework, disorder-specific formulations and manuals have been developed to enhance treatment specificity and relevance to patients (11).

In the metacognitive model of OCD (11, 15), two distinct and specific types of metacognitive beliefs are proposed: 1) thought-fusion beliefs, which concern the meaning and consequences of intrusive thoughts and spontaneous inner events (e.g., feelings), and 2) beliefs about rituals, which concern the advantages of performing rituals and neutralizing behaviors, as well as internal criteria for when it is safe to stop (i.e., stop signals). These beliefs are hypothesized to guide the individual’s interpretation and response to thoughts, feelings, or doubts, hence activating maladaptive self-regulation strategies (i.e., CAS). In OCD patients, CAS strategies typically comprise of excessive thinking (e.g., worry, rumination, and doubting), threat-monitoring (e.g., strategic attention directed towards contamination, unwanted thoughts, memory gaps, etc.), and maladaptive coping behaviors or rituals (e.g., magical sayings, repeating actions, reassurance seeking, etc.). Consequently, there are fundamental differences between the metacognitive model and other cognitive models, leading to substantial differences in how OCD is formulated and treated.

MCT targets specific components of metacognition and mental regulation strategies (e.g., through verbal reattribution, behavioral experiments, and practicing alternative ways of responding to triggers) and does not involve reality testing of cognition or prolonged exposure (11) that are common features of CBT for pediatric OCD. In the metacognitive model, biases in cognitions or appraisals targeted in CBT (e.g., inflated responsibility or overestimation of threat) are considered products of biases in metacognition. Thus, interventions targeted at lower-level cognition are considered unnecessary and may hinder optimal treatment response. Guided by the metacognitive model, MCT therefore exclusively focuses on 1) modifying metacognitive beliefs and goals for mental regulation, and 2) changing how one relates and responds to thoughts and spontaneous inner events.

In adults, three randomized controlled trials have compared individual MCT and ERP for OCD demonstrating significant and large effects for both treatments (16–18). Two uncontrolled trials have also evaluated group MCT for OCD. In a pilot study, Rees and van Koesveld (19) evaluated group MCT in an outpatient setting and reported that seven out of eight patients were recovered at 3-month follow-up. Papageorgiou et al. (20) evaluated group MCT using CBT as a benchmark and found that both treatments were associated with significant improvements in both primary and secondary symptoms, but that the MCT group had a significantly higher response rate (MCT: 86%, CBT: 64%). Interestingly, a recent systematic review suggests that metacognitive change (not specific to OCD) is a more important correlate of symptom improvements in patients treated for OCD with CBT/ERP compared to change in cognitive beliefs (21). Moreover, several studies also indicate significant pre- to post-treatment reductions in OCD-specific metacognitive beliefs following MCT for OCD (18, 22–24), observations that align with the S-REF predictions and are indicative of the theorized mechanisms of change in MCT.

To date, only two studies have evaluated MCT for children with OCD. First, Simons et al. (25) conducted a case-series with random allocation to MCT or ERP (*N* = 11, age: 8-17) and demonstrated significant and large post-treatment effects for both treatments (ERP: *g* = 2.2; MCT: *g* = 2.9) which were maintained at 2-year follow-up. More recently, a pilot study of group MCT for pediatric OCD (*N* = 11, age: 11-17; 26) also showed significant and large reductions in OCD symptoms (CY-BOCS, *g* = 1.90) and response and remission rates of 82% and 55%, respectively. Additionally, large improvements were found in secondary symptoms of anxiety and depression, and trait-worry (*g*s = 0.78 to 1.21), as well as metacognitive beliefs (*g* = 1.10). Preliminary data also suggested a positive relationship between trajectories of change in OCD distress and OCD-specific metacognitive strategies and beliefs, suggesting that, in line with the S-REF model, metacognitive change could be a potential mediator of treatment effects (26). Only eight group sessions were delivered in the study by Reinholdt-Dunne et al. (26), indicating that MCT can be effectively delivered in a group format and with relatively few therapist resources. The group format may even contribute to normalization of obsessions and facilitate metacognitive change (e.g., through experience sharing when testing thought-fusions and practicing new ways of relating to triggers). In sum, there is preliminary evidence suggesting that MCT for pediatric OCD could be a promising alternative to current gold standard treatments. However, no study to date has explored whether the previous findings generalize to samples with high rates of comorbid disorders. As a majority of children with OCD present with comorbidity (4), this is nevertheless an important step in evaluating the feasibility of MCT for pediatric OCD in naturalistic settings.

Based on an uncontrolled study design, we aimed to investigate the magnitude of effects, response and remission rates, and trajectories of change associated with group MCT for pediatric OCD in a sample of 37 children and adolescents with and without comorbid disorders treated in a naturalistic setting. It was hypothesized that MCT would be associated with large improvements in OCD symptoms, comorbid symptoms (i.e., anxiety and depression), trait-worry, and metacognitions from pre- to post-treatment. Further, consistent with the key therapeutic targets in MCT, large reductions were expected in OCD-specific metacognitive strategies (i.e., CAS) and beliefs (i.e., fusion beliefs and beliefs about rituals) measured at four time points from session 1 through 8. In addition, we aimed to provisionally explore response rates and patterns of change based on three diagnostic subgroups: a subsample with OCD only (*n* = 15), one with OCD and comorbid neurodevelopmental disorders (*n* = 12), and one with OCD and comorbid anxiety disorders (*n* = 10). We were interested in evaluating changes in symptoms and metacognitive mechanisms associated with group MCT in light of comorbidity.

## 2 Methods

### 2.1 Participants

Children were included if they a) were between 8–17 years of age, b) had a primary disorder of OCD as defined in the Diagnostic and Statistical Manual of Mental Disorders, fourth edition (DSM-IV; 27), c) presented with OCD symptom severity ≥ 16 measured by the Children’s Yale-Brown Obsessive Compulsive Scale (CY-BOCS; 28). Excluding criteria were a) meeting diagnostic criteria for another psychiatric disorder with higher treatment priority (e.g., psychotic symptoms or severe depression), b) use of medication not monitored by health professionals, c) change in dose of psychotropic medication in the past three months, and d) receiving concurrent psychological treatment.

### 2.2 Measures

#### 2.2.1 Diagnostic assessment

*Anxiety Disorders Interview Schedule – Child Version (ADIS-IV-C;* 29*).* The ADIS-C is a clinician-administered, semi-structured diagnostic interview used to assess the presence and severity of child anxiety and related disorders according to DSM-IV. While an updated version of the DSM exists (i.e., DSM-5), a fifth version of the ADIS was not published until recently (30) and is not yet available in Danish. The ADIS (child/parent versions) has demonstrated good psychometric properties (31) and is one of the most commonly used diagnostic interviews in children (32). In the present study, diagnoses were obtained based on child report.

#### 2.2.2 Symptom measures

*Children’s Yale-Brown Obsessive Compulsive Scale* (*CY-BOCS;* 28). The CY-BOCS is a clinician-administered, semi-structured interview conducted to assess the content and severity of OCD symptoms. It consists of 10 items assessing the severity of obsessions and compulsions on a 5-point scale from 0 (“none”) to 4 (“extreme”), with total scores ranging from 0 to 40. The CY-BOCS has shown good psychometric properties (28, 33) and is considered the gold standard for assessing OCD symptoms and quantifying symptom changes over time (2, 34). In the current study, the CY-BOCS was conducted with the child only.

*Revised Anxiety and Depression Scale – Child and Parent Versions (RCADS;* 35*).* The RCADS is a 47-item self-report measure assessing symptoms of anxiety and depression according to the DSM-IV criteria. It consists of six subscales, including separation anxiety, social phobia, generalized anxiety, OCD, panic disorder, and major depressive disorder. Items are rated on a 4-point scale from 0 (“never”) to 3 (“always”), with total scores ranging from 0-141. The scale has shown good psychometric properties (e.g., 36, 37). In the present study, both child and parent versions of the scale were administered. Cronbach’s *α* for the total scales were .91 to .95.

*Penn State Worry Questionnaire for Children (PSWQ-C;* 38*)*. The PSWQ-C is a 14-item self-report measure assessing levels of worry, with items rated on a 4-point scale from 0 (“never true”) to 3 (“always true”). After recoding reverse items, total scores range from 0-42, with higher scores indicating greater levels of worry. The scale has demonstrated strong psychometric properties (e.g., 38; 39). In the present sample, Cronbach’s *α* was .81.

#### 2.2.3 Metacognitive beliefs and strategies

*Metacognitions Questionnaire for Children – 30-Item Version*(*MCQ-C_30_*; 40). The MCQ-C_30_ is a 30-item self-report measure assessing metacognitive beliefs in children and adolescents. Based on five subscales, it measures positive beliefs about worry, negative beliefs about uncontrollability and danger of worry, cognitive confidence, need for control, and cognitive self-consciousness. Items are rated on a 4-point scale from 1 (“do not agree”) to 4 (“agree very much”). The scale is based on the Metacognitions Questionnaire for Adolescents (MCQ-A; 41) with wordings modified for use with children. It was originally adapted in the German language (40) and has subsequently been translated and tested in a Danish context, showing good internal consistency (*α* = .87) for the total scale (42). In the present sample, Cronbach’s *α* was .90.

*The Obsessive-Compulsive Disorder Scale (OCD-S;* 11*).* The OCD-S is a 26-item clinical tool used to monitor changes in OCD distress and OCD-specific metacognitive strategies and beliefs. Item 1 measures distress and impairment related to obsessive thoughts and urges experienced in the past week and is scored on a scale from 0 (“not at all”) to 8 (“extremely - the worst they have ever been”). Items 2A-2I and 3A-3F measure maladaptive coping and avoidance strategies (i.e., CAS) and is scored on a scale from 0 (“none of the time”) to 8 (“all of the time”). Items 4A to 4J measure metacognitive beliefs and are rated on a scale from 0 (“I do not believe this at all”) to 100 (“I’m completely convinced this is true”). More specifically, items 4A to 4E measure fusion beliefs (e.g., “Obsessional thoughts could change me as a person”), and items 4F to 4J measure beliefs about rituals (e.g., “I cannot have a peace of mind unless I perform my rituals”). For the purpose of the present study, the wording of some items was only slightly simplified for use with children and young people. Cronbach’s *α* was .85 for CAS, .69 for fusion beliefs, and .79 for beliefs about rituals.

### 2.3 Design and procedure

Participating families were self-referred for treatment at a private practice in Copenhagen, specialized in child and youth mental health problems. The treatment was advertised on the clinic’s website. Interested families were invited to two 120-minute physical meetings with an experienced licensed clinical psychologist. Parents/guardians gave written informed consent to their child’s participation in the treatment as well as to the data being used for research purposes. Oral assent was obtained from all children and written informed consent was obtained when ≥ 16 years of age. In the first meeting, anamnestic information was obtained from parents/guardians and a diagnostic interview was conducted with the child based on the ADIS-C. In the second meeting (child only), the CY-BOCS was administered and an individual metacognitive case formulation for OCD (11) was conceptualized. The group treatment started continuously from January 2022 to March 2024 and participants were allocated to a group based on the time of their inquiry (i.e., regardless of comorbid presentations). Approximately one week following treatment completion, children were invited for a 2-hour diagnostic interview where the ADIS-C and CY-BOCS were administered. Concerning self-report measures, RCADS (child and parent versions), PSWQ-C, and MCQ-C_30_ were administered at pre- and post-treatment, and OCD-S was administered in the beginning of session 1, 3, 6, and 8.

### 2.4 Treatment

In total, 10 groups were held with 2–7 children in each. The treatment consisted of eight weekly 75-minute group sessions which followed the MCT treatment principles for OCD in adults (11). The session-by-session content has been described in detail elsewhere (26). Parents were offered a 1-hour workshop prior to session 1 and again at mid-treatment (i.e., between session 4 and 5). They were informed about OCD and socialized to the treatment rationale (workshop 1) and how best to support their child (workshop 2). All group sessions and parent workshops were delivered by the first author, who is a licensed clinical psychologist with formal training in MCT (MCT institute^®^ level 2 diploma). Seven groups were assisted by a master’s student in psychology and/or a clinical psychologist who either held a MCT institute^®^ level 1 or 2 diploma or was undergoing formal MCT training. If participants were unable to attend a group session, they were offered an individual 1-hour compensatory session by the assistant. Supervision was provided by the first author every other week. Additionally, treatment progress (or lack thereof) was discussed among therapists on an ad hoc basis either informally or as part of the clinic’s regular meetings.

### 2.5 Statistical analyses

All analyses were performed in STATA IC version 19.5. First, the effect of time on CY-BOCS (primary outcome), RCADS child and parent versions, PSWQ-C, and MCQ-C_30_ (secondary outcomes) were assessed using planned contrasts within multilevel modelling (MLM), which allows for estimation of changes in repeated measures over time despite missing data (43). However, there was no missing data on any self-report (from the children) or clinician-based measure, and parent report of child symptoms (RCADS) was obtained from at least one parent for 32 participants at pre-treatment (32 mothers and 29 fathers) and 34 participants at post-treatment (34 mothers and 26 fathers).

Analyses were conducted using linear mixed-effects models estimated via maximum likelihood (ML). This approach allows inclusion of participants with partially missing repeated measures under the assumption that data are missing at random (MAR). We assessed the assumption of missing completely at random (MCAR) using Little’s test *χ^2^*(14) = 21.16, *p* = .10. The test indicated that missingness was not systematically related to observed data, supporting the use of likelihood-based mixed models under the assumption of missing at random (MAR). Thus, all available observations contributed to parameter estimation.

Next, we used random coefficients models to investigate change trajectories on the OCD-S (i.e., OCD distress, CAS strategies, fusion beliefs, and beliefs about rituals) measured at four time points (i.e., session 1, 3, 6, and 8). We computed a time effect, representing change over time. The linear combination of coefficients function was used to create model-predicted means and all planned contrasts. Effect sizes were expressed as Hedges’ *g* which is recommended in smaller samples, with values of 0.2, 0.5, and 0.8 considered small, medium, and large (44).

Response and remission rates were defined using international expert consensus criteria (45) and calculated based on participants’ CY-BOCS total scores at pre- and post-treatment. *Remission* was defined by a CY-BOCS score of ≤ 12 plus a Clinical Global Impression – Severity (CGI-S) rating of “normal, not ill at all”, or “borderline mentally ill”, lasting for at least one week. Treatment *response* was defined as a ≥ 35% reduction in CY-BOCS score plus Clinical Global Impression – Improvement (CGI-I) rating of “very much improved” or “much improved” lasting for at least one week. *Partial response* was defined as a ≥ 25% but < 35% reduction in CY-BOCS score plus CGI-I rating of at least “minimally improved” lasting for at least one week, and *no response* was defined as < 25% reduction in CY-BOCS score.

To explore response rates and patterns of change based on comorbid presentations, participants were split into three diagnostic groups: 1) OCD only, 2) OCD with comorbid neurodevelopmental disorder(s), that is, attention deficit hyperactivity disorder (ADHD) and autism spectrum disorder (ASD), and 3) OCD with comorbid anxiety disorder(s). If participants presented with both comorbid neurodevelopmental and anxiety disorders, they were categorized based on the former.

## 3 Results

### 3.1 Descriptive statistics

#### 3.1.1 Participants

A total of 37 children and adolescents (72.97% females, *n* = 27) between 9 and 17 years of age (*M* = 12.35 ± 2.49) were included in the study. The participants were predominantly of Danish ethnicity, resided in Copenhagen or the surrounding suburbs, and either paid for treatment or had it covered by their health insurance or municipality. Fifteen participants presented with OCD only, while a majority presented with comorbid neurodevelopmental disorders (ADHD, *n* = 10; ASD, *n* = 3) and/or anxiety disorders (*n* = 12). Three participants presented with both comorbid neurodevelopmental and either anxiety disorder(s) (*n* = 2) or a depressive disorder (*n* = 1). Diagnoses for the total sample and diagnostic subgroups are displayed in **Table 1**. No participants dropped out of treatment and all participants attended at least seven out of eight group sessions. No more than one individual compensatory session was delivered per group.

**Table 1:** Diagnoses at pre- and post-treatment based on the ADIS-C.

Diagnoses	Total sample (*N* = 37)		OCD only (*n* = 15)		OCD with ADHD/ASD (*n* = 12)		OCD with anxiety (*n* = 10)	
	Pre	Post	Pre	Post	Pre	Post	Pre	Post
OCD	37	6	15	1	12	1	10	4
Anxiety disorders								
Separation anxiety	2	0	–	–	0	–	2	0
Social anxiety	1	0	–	–	0	–	1	0
Panic disorder	1	0			0	–	1	0
Specific phobia	2	1	–	–	1	0	1	1
Generalized anxiety	12	4	–	–	2	0	10	4
Depressive disorders								
Dysthymia	1	0	–	–	1	0	0	–

### 3.2 Effects associated with treatment

Group MCT for pediatric OCD was associated with significant and large improvements in OCD symptoms (CY-BOCS), anxiety and depression (RCADS child and parent report), and metacognitive beliefs (MCQ-C_30_) from pre- to post-treatment (*p*s <.001, *g*s = 0.88 to 2.25). A significant and moderate effect (*p* <.001, *g* = 0.71) was also found for trait-worry (PSWQ-C). Within each of the three diagnostic subgroups, improvements in pre- to post-treatment scores were observed across both primary and secondary outcome measures. **Table 2** presents model predicted means and *SE* values for the total sample and subgroups, and the effect of time for the total sample.

**Table 2:** Model predicted means showing change over time from pre- to post-treatment in the total ITT sample.

Outcome/sample	Model predicted means ITT				
	Pre-MCT *M* (*SE*)	Post-MCT *M* (*SE*)	Change over time *Z*-value (*p*-value)	Time effect 95% CI	Hedges’ *g*
CY-BOCS	26.78 (.77)	11.97 (.93)	-13.97 (0.000)	[-16.89, -12.73]	2.25
Gr1	27.38 (1.22)	11.33 (1.46)			
Gr2	25.92 (1.33)	12.33 (1.63)			
Gr3	27.00 (1.45)	12.50 (1.79)			
RCADS_child_	57.16 (3.96)	37.78 (3.96)	-5.51 (0.000)	[-26.27, -12.49]	0.88
Gr1	54.00 (5.67)	28.73 (5.67)			
Gr2	46.75 (6.34)	38.50 (6.34)			
Gr3	74.40 (6.94)	50.50 (6.94)			
PSWQ-C	26.59 (1.42)	20.89 (1.42)	-4.45 (0.000)	[-8.21, -3.19]	0.71
Gr1	25.67 (2.00)	18.80 (2.00)			
Gr2	22.00 (2.24)	19.25 (2.23)			
Gr3	33.50 (2.45)	26.00 (2.45)			
MCQ-C_30_	33.41 (1.97)	20.59 (1.97)	-5.71 (0.000)	[-17.20, -8.42]	0.91
Gr1	31.73 (2.95)	16.73 (2.95)			
Gr2	31.42 (3.30)	20.08 (3.30)			
Gr3	38.30 (3.62)	27.00 (3.62)			
RCADS_mother_	58.44 (3.52)	39.24 (3.44)	-5.71 (0.000)	[-25.79, -12.62]	0.98
Gr1	64.90 (5.37)	37.67 (5.13)			
Gr2	52.89 (6.17)	39.52 (5.99)			
Gr3	55.56 (6.61)	41.58 (6.86)			
RCADS_father_	50.98 (3.73)	34.16 (3.85)	-5.14 (0.000)	[-23.24, -10.41]	0.97
Gr1	57.10 (5.43)	32.62 (5.54)			
Gr2	47.18 (6.48)	36.51 (6.48)			
Gr3	44.71 (7.54)	34.57 (8.23)			

Gr1, OCD only; Gr2, OCD with comorbid neurodevelopmental disorder(s); Gr3, OCD with comorbid anxiety disorder(s); CY-BOCS, Children’s Yale-Brown Obsessive Compulsive Scale; MCQ-C_30_, Metacognitions Questionnaire for Children - 30-Item Version; PSWQ-C, Penn State Worry Questionnaire for Children; RCADS, Revised Children’s Anxiety and Depression Scale (child and parent versions).

### 3.3 Response and remission rates, and change in diagnostic status

Response and remission rates were calculated based on international expert consensus criteria (45) and are presented in **Table 3**. For the total sample, 30 participants (81.1%) were classified as responders, out of which 19 (51.4%) classified as being in remission. Additionally, three participants (8.1%) classified as partial responders, whilst four (10.8%) were non-responders. When exploring levels of treatment response based on diagnostic subgroups, the three partial responders belonged to the subgroup with comorbid ADHD/ASD and the four non-responders belonged to the subgroup with comorbid anxiety disorders.

**Table 3:** Treatment response and remission based on CY-BOCS pre- and post-treatment scores for the total sample and diagnostic subgroups.

Sample	Remission *n*	Response *n*	Partial response *n*	No response *n*
Total sample (*N* = 37)	19	30	3	4
OCD only (*n* = 15)	9	15	–	–
OCD w. ADHD/ASD (*n* = 12)	4	9	3	–
OCD w. anxiety (*n* = 10)	6	6	–	4

Based on international expert consensus criteria for OCD (45).

In terms of changes in diagnoses according to the clinical assessment (ADIS-C), 31 out of 37 participants (83.8%) no longer met diagnostic criteria for OCD at post-treatment. Within subgroups, this was the case for 14 out of 15 participants with OCD only, 11 out of 12 participants with OCD and ADHD/ASD, and six out of 10 participants with OCD and anxiety disorders. **Table 1** displays diagnoses at pre- and post-treatment based on ADIS-C. In the total sample, eight out of 12 participants also no longer met criteria for generalized anxiety disorder at post-treatment. Additionally, two out of two participants no longer met criteria for separation anxiety, one out of two no longer met criteria for specific phobia, and the ones with social anxiety, panic disorder, and dysthymia no longer met diagnostic criteria for these disorders at post-treatment.

### 3.4 Trajectories of change in OCD distress and proposed mechanisms

For the total sample, results showed a significant and large change over time in OCD distress, CAS strategies, and OCD-specific metacognitive beliefs (fusion beliefs and beliefs about rituals) measured by the OCD-S at four time points from session 1 through 8 (*p*s <.001, *g*s = 0.90 to 1.67). In all three subgroups, mean scores similarly indicated improvements in OCD distress and metacognitive mechanisms from session 1 to 8. However, numerical differences in the pattern of change were observed for fusion beliefs. Whereas the subgroup with OCD only displayed a continuous improvement over time, the subgroup with comorbid ADHD/ASF displayed relatively stable fusion beliefs from session 1-6, followed by a steep improvement from session 6-8. Also, the subgroup with comorbid anxiety displayed a worsening in fusion beliefs from session 1-3, followed by steep improvements from session 3-8. **Table 4** presents model predicted means and *SE* values for the total sample and subgroups, and the effect of time from session 1 through 8 for the total sample.

**Table 4:** Model predicted means showing change over time for OCD-S from session 1 through 8 in the total ITT sample.

Outcome/sample	Model predicted means ITT						
	Session 1 *M* (*SE*)	Session 3 *M* (*SE*)	Session 6 *M* (*SE*)	Session 8 *M* (*SE*)	Change over time Z-value (*p*-value)	Time effect 95% CI	Hedges’ *g*
OCD distress	5.27 (.23)	5.38 (.23)	4.03 (.24)	2.39 (.25)	-11.22 (0.000)	[-1.17, -.82]	1.56
Gr1	5.13 (.35)	5.40 (.36)	3.87 (.37)	2.60 (.40)			
Gr2	5.50 (.40)	5.42 (.40)	4.00 (.42)	2.17 (.44)			
Gr3	5.20 (.36)	5.30 (.44)	4.30 (.46)	2.35 (.48)			
CAS	3.61 (.23)	3.50 (.24)	2.87 (.24)	1.82 (.25)	-9.04 (0.000)	[-.64, -.41]	1.24
Gr1	3.59 (.37)	3.73 (.37)	3.01 (.38)	2.21 (.39)			
Gr2	3.51 (.41)	3.15 (.42)	2.73 (.42)	1.58 (.44)			
Gr3	3.75 (.45)	3.58 (.46)	2.83 (.46)	1.52 (.48)			
Fusion beliefs	144.28 (12.56)	147.05 (12.56)	90.51 (12.56)	54.41 (12.56)	-7.15 (0.000)	[-40.53, -23.10]	0.90
Gr1	152.73 (19.07)	129.80 (19.07)	66.33 (19.07)	48.07 (19.07)			
Gr2	126.67 (21.32)	134.17 (21.32)	119.17 (21.32)	54.33 (21.32)			
Gr3	152.72 (23.35)	188.40 (23.35)	122.00 (23.35)	64.02 (23.35)			
Beliefs about rituals	286.16 (17.01)	230.32 (17.01)	155.73 (17.01)	95.28 (17.01)	-11.67 (0.000)	[-75.59, -53.86]	1.67
Gr1	293.07 (26.44)	233.00 (26.44)	135.40 (26.44)	81.47 (26.44)			
Gr2	267.50 (33.82)	210.83 (33.82)	165.83 (33.82)	97.75 (33.82)			
Gr3	298.21 (27.89)	249.70 (28.45)	174.10 (30.09)	113.02 (32.63)			

Gr1, OCD only; Gr2, OCD with comorbid neurodevelopmental disorder(s); Gr3, OCD with comorbid anxiety disorder(s).

## 4 Discussion

The present study reports on treatment effects, response and remission rates, and trajectories of change associated with group MCT for pediatric OCD in light of comorbid neurodevelopmental and anxiety disorders.

Overall, results showed that group MCT was associated with large effects on measures of primary OCD symptoms (CY-BOCS, *g* = 2.25), comorbid symptoms of anxiety and depression reported by both children and parents (RCADS, *g*s = 0.88 to 0.98), and metacognitive beliefs (MCQ-C_30_, *g* = 0.91), as well as a moderate effect on trait worry (PSWQ-C, *g* = 0.71). While no direct comparisons can be made, the effect observed on CY-BOCS in the present study is similar to the within-group effect for CBT interventions (*g* = 2.43) and substantially higher than the effect for waitlist controls (*g* = 0.22) reported in a meta-analysis by Öst et al. (6). Moreover, based on international consensus criteria (45), response and remission rates for the total sample were 81.1% and 51.4%, respectively, and clinical assessments based on the ADIS-C showed that 83.8% no longer met diagnostic criteria for OCD at post-treatment. These numbers are similar to the response and remission rates for CBT interventions (69.6% and 52.7%) and substantially higher than the rates for waitlist controls (13.0% and 10.1%) reported by Öst et al. (6). Using this as a benchmark guide for treatment effects, our results are encouraging in suggesting that group MCT for pediatric OCD is a promising and resource efficient treatment alternative, also in samples with high rates of comorbid disorders.

Although the treatment was directed at modifying OCD-specific metacognitive strategies and beliefs, a majority of participants also remitted from their comorbid disorders according to the clinical assessments at post-treatment. In the total sample, eight out of 12 participants no longer met criteria for GAD, two out of two participants no longer met criteria for separation anxiety, one out of two no longer met criteria for specific phobia, and the individuals with social anxiety, panic disorder, and dysthymia also no longer met diagnostic criteria for these disorders at post-treatment. In sum, the sample as a whole met diagnostic criteria for 19 comorbid anxiety and depressive disorders at pre-treatment, out of which only five remained following treatment. This indicates that MCT may also be effective in treating emotional disorders comorbid to pediatric OCD, hence supporting the transdiagnosti nature of MCT (11). This notion is further supported by the large reduction found in generic metacognitive beliefs assessed by the MCQ-C_30_. One explanation could be that the disorder-specific MCT formulations and treatments share a common set of goals and interventions, such as facilitating *detached mindfulness* (11) as an alternative way of relating to mental events (e.g., obsessional thoughts and trigger thoughts more generally) that facilitate correction of biases in the metacognitive control system including OCD-specific metacognitive beliefs (e.g., “thoughts can cause accidents”) and more universal dysfunctional metacognitive beliefs (e.g., “worrying is uncontrollable”) (12).

Concerning trajectories of change, results showed large improvements in OCD-specific distress (*g* = 1.56), CAS strategies (*g* = 1.24), though-fusion beliefs (*g* = 0.90), and beliefs about rituals (*g* = 1.67) measured at four time points from session 1 through 8. Moreover, reductions in distress occurred in parallel to metacognitive change, consistent with a positive relationship between these variables. These findings support our hypothesis and provide further support for the S-REF model in suggesting that metacognitive strategies and beliefs could be potential mediators of treatment effects. An important next step will be to test these relationships and their proposed directionality statistically. Such relationships have been demonstrated in an open trial of group MCT for children and adolescents with anxiety and depressive disorders, showing that changes in metacognitive strategies and beliefs (measured by the CAS-1 and Metacognitions Questionnaire - Adolescent Version) temporally preceded changes in symptoms within individuals (46). Nevertheless, in testing the proposed relationship between distress and OCD-specific metacognitive beliefs, it may be necessary to separate different types of fusion beliefs. For instance, the OCD-S measures both thought-event fusions (e.g., “Obsessional thoughts increase the chance of negative events in the future”) and thought-action fusions (e.g., “Obsessional thoughts could make me do bad things), and individuals with OCD may score high on only one or few of these, whilst scoring low on others. Consequently, the mean score of this subscale could potentially mask specific metacognitive beliefs central to the individual’s CAS and symptoms. Hence, differentiating between types of fusion beliefs may be an important step in testing OCD-specific metacognitions as potential mechanisms of change.

When separating participants in the present study based on diagnostic presentations, improvements were observed in all three subgroups across symptom- and mechanism measures. However, based on Mataix-Cols et al.’s (45) definition of a clinically significant treatment response, slightly lower response rates were observed in the two subgroups with comorbid disorders. Whereas all participants with OCD only classified as responders, three out of 12 participants with comorbid neurodevelopmental disorders classified as partial responders, and four out of 10 participants with comorbid anxiety disorders classified as non-responders. However, based on the ADIS-C, 11 out of 12 participants with comorbid neurodevelopmental disorders no longer met diagnostic criteria for OCD, indicating an overall favorable treatment response similar to participants with OCD only. Nevertheless, for the subgroup with anxiety disorders, clinical assessments revealed that the four non-responders still met diagnostic criteria for both OCD and GAD at post-treatment.

One explanation for the observed differences in response rates may be found in the pattern of metacognitive change. When inspecting mean scores from session 1 through 8, the data indicated a continuous and uninterrupted improvement in thought-fusion beliefs for the subgroup with OCD only, whereas mean scores indicated a delayed, but steeper improvement in fusion beliefs for the two subgroups with comorbid disorders. Specifically, for the subgroup with ADHD/ASF, level of fusion beliefs remained largely the same from session 1 through 6 and then rapidly improved in the later stage of treatment (session 6-8). For the subgroup with anxiety disorders, mean scores indicated a worsening in fusion beliefs in the early stage of treatment (session 1-3), followed by steep improvements throughout the mid and later stages of treatment (sessions 3-8). If replicated and confirmed statistically in future studies, it is possible that such differences in metacognitive change trajectories may be explained by individual differences in the awareness of holding specific metacognitive beliefs, which may correlate with comorbidity/complexity. Delayed or slower patterns of change observed in subgroups with comorbidity could be due to an increasing awareness of the existence of such beliefs in the early stage of treatment (e.g., in response to being socialized to the metacognitive model), whereas those with less complex presentations may have an easier access to their metacognitive beliefs, enabling a more rapid change.

In sum, the findings from the present study extends the previous literature (25, 26) by suggesting that MCT for pediatric OCD is a feasible treatment in samples with different diagnostic presentations. This has important clinical implications as no less than 64% of children with OCD present with comorbidity (4), which was also reflected in the current sample (63%). In turn, the present study provides preliminary support for the applicability of MCT for pediatric OCD in a naturalistic setting. Moreover, our findings suggest that comorbid disorders, including neurodiversity, do not necessarily prevent children from benefitting from MCT delivered in a group format. While our results did indicate slightly differing response rates across types of comorbid presentations, they also suggested that lower response rates may be associated with delayed patterns of change in thought-fusion beliefs. Thus, monitoring changes in metacognitive mechanisms (e.g., thought-fusion beliefs) over the course of MCT may offer valuable clues about treatment targets and trajectories of change which likely are linked to treatment response.

### 4.1 Limitations and future directions

Several important limitations must be acknowledged. First, the uncontrolled design means that we cannot partial out the effect of time and spontaneous recovery on outcomes. The findings from the present study should therefore be considered preliminary, although providing an important step in evaluating MCT for pediatric OCD in light of comorbid disorders. Future studies are encouraged to evaluate MCT for pediatric OCD both with and without comorbid disorders using more stringent designs. Second, the sample size was small for the three diagnostic subgroups and therefore, future studies will need to test and confirm our findings statistically in larger samples. Third, the first author led all group sessions and parental workshops and therefore, we cannot rule out any therapist effects. Fourth, participants were self-referred for treatment at a private practice in Copenhagen and although some families had the treatment covered by their health insurance or municipality, participants were most likely more resourceful and of higher educational background than country average. Hence, future studies are encouraged to examine the influence of demographics, and whether the findings generalize to community settings and more rural areas. Nevertheless, the present study indicates that MCT for pediatric OCD is a promising and cost-effective treatment and provides support for its applicability in real-world clinical settings. Finally, due to lack of follow-up assessments, it is unclear whether treatment gains were maintained following treatment completion. While the case study by Simons et al. (25) showed that the effects associated with MCT were upheld through a 2-year follow-up period, future studies are encouraged to evaluate both short- and long-term effects of MCT for pediatric OCD.

## 5 Conclusions

The present study preliminary indicates that group MCT is a promising treatment for pediatric OCD across different diagnostic presentations, demonstrated by large improvements in symptoms and metacognitive mechanisms as well as favorable response rates. However, slightly lower response rates were observed in the two subgroups with comorbid disorders, accompanied by indications of delayed, yet steeper trajectories of change in thought-fusion beliefs. We note that these latter findings must be interpreted with caution given the small sample and thus, should be confirmed statistically in larger, more diverse samples using more stringent study designs. Nonetheless, our findings are encouraging in suggesting that group MCT is a feasible treatment for pediatric OCD in real-world clinical settings and that metacognitive change patterns could provide useful information about differing response rates and individual adjustments.

## Data Availability

The dataset presented in this article are not readily available because data is confidential. Requests to access the datasets should be directed to MR-D, kontakt@metaminds.dk.
